# Utility of the ECG-Based Simple Score for Predicting Post-Stroke Atrial Fibrillation in a Real-World Clinical Setting

**DOI:** 10.1177/10760296251361083

**Published:** 2025-07-24

**Authors:** Yusuf Hosoglu, Ayse Hosoglu, Veysi Kavalcı, Hakan Tibilli, Sezer Markirt, Erman Altınışık

**Affiliations:** 1 599324 Private Koru Sincan Hospital, Ankara, Turkey; 2 Başkent University Ankara Hospital, Ankara, Turkey; 3 162296 Adiyaman Universitesi, Adıyaman, Turkey; 4 University of Health Sciences, Gaziantep City Hospital, Gaziantep, Turkey

**Keywords:** atrial fibrillation, Ischemic stroke, ECG risk score, SIMP3L2E score, supraventricular ectopy, Holter monitoring, stroke recurrence

## Abstract

**Background:**

Atrial fibrillation (AF) significantly increases the risk of ischemic stroke and often remains asymptomatic until stroke onset. Identifying stroke survivors at high risk for incident AF is critical for targeted anticoagulation therapy. This study aimed to evaluate the predictive utility of the ECG-based SIMP3L2E score alone and combined with Holter-detected supraventricular ectopy for incident AF and recurrent stroke in ischemic stroke survivors.

**Methods:**

This prospective observational study enrolled 77 patients hospitalized with acute ischemic stroke between January and September 2021. Although the SIMP3L2E score was published in 2024, all required ECG parameters were collected prospectively during initial hospitalization and retrospectively calculated for analysis. Incident AF and recurrent strokes were assessed retrospectively over a three-year follow-up. Predictive performance was evaluated using logistic regression and receiver operating characteristic (ROC) analysis.

**Results:**

Of the participants, 27 (35.1%) had high SIMP3L2E scores (≥12). Incident AF occurred in 12 patients (15.6%) and recurrent stroke in 9 patients (11.7%). The SIMP3L2E score alone had modest predictive ability (AUC = 0.588). However, supraventricular ectopy detected by Holter was independently predictive of incident AF (OR: 0.092; 95% CI: 0.016-0.538; p = .008) and significantly improved predictive accuracy (AUC = 0.797).

**Conclusion:**

The SIMP3L2E ECG score demonstrated limited predictive power alone in older post-stroke patients but showed substantially improved discrimination when combined with Holter-detected supraventricular ectopy. Integrating static ECG scores with dynamic rhythm monitoring could enhance risk stratification for incident AF following ischemic stroke. Future studies should validate these findings in larger, diverse populations.

## Introduction

Stroke is a leading cause of death and long-term disability, with recurrence rates reaching 5.7% within the first year and up to 22.5% within five years.^1,2^ Atrial fibrillation (AF), the most common sustained arrhythmia, increases the risk of ischemic stroke nearly five-fold and is frequently asymptomatic, remaining undiagnosed until after stroke onset in many cases.^3^ Importantly, up to 27% of AF cases remain asymptomatic and often go undiagnosed until a stroke occurs.^4^ Identifying stroke survivors at high risk for incident AF is therefore critical for guiding timely anticoagulation and reducing recurrence. While several ECG features have shown potential for AF prediction,^5,6^ the SIMP3L2E score, a simple tool derived from six ECG parameters, has not yet been validated in older, post-stroke populations.^7^ This study aimed to evaluate the predictive utility of the SIMP3L2E score (both alone and in combination with supraventricular ectopy detected on Holter ECG) for predicting incident AF and recurrent stroke during a three-year follow-up in a prospective cohort of patients hospitalized with acute ischemic stroke.

Although clinical scores such as CHADS₂, CHA₂DS₂-VASc, and AS5F have been proposed to predict post-stroke AF, their performance is limited and they typically omit ECG-based markers.^8,9^ In contrast, the SIMP3L2E score, which demonstrated good discrimination in a general middle-aged cohort, relies solely on ECG parameters that are routinely collected during hospitalization for stroke.^7^ Because stroke patients often undergo Holter monitoring during workup, combining this static ECG score with dynamic rhythm findings may improve AF risk stratification. This study therefore explores a practical method for enhanced AF prediction in a real-world clinical setting.

## Methods

### Study Design and Population

This prospective observational cohort study included patients hospitalized with acute ischemic stroke at a tertiary care hospital between January and September 2021. The study protocol was approved by the institutional ethics committee (approval date: December 22, 2020; decision number: 2020/11-18) and conducted in accordance with the principles of the Declaration of Helsinki. Written informed consent was obtained from all participants prior to enrollment.

Of 147 consecutive patients initially screened, 5 patients who declined to provide informed consent were excluded before eligibility assessment. The remaining 142 patients were evaluated based on predefined clinical and diagnostic criteria. Patients were excluded if they had a known history of AF (n = 20), heart failure with reduced LVEF (<40%) (n = 10), or baseline serum creatinine >1.5 mg/dL (n = 8). Further exclusions included unavailable or uninterpretable baseline 12-lead or Holter ECG recordings (n = 12), or the presence of baseline rhythm abnormalities or conduction disturbances that precluded ECG interval measurement-such as atrial flutter, supraventricular tachycardia, bradycardia (heart rate <50 bpm), tachycardia (>120 bpm), paced rhythms, or bundle branch blocks (n = 17). Three additional patients were excluded due to known thyroid disease or abnormal thyroid function tests (TSH <0.4 or >4.5 µIU/mL or free T4 outside the institutional reference range).

**Figure 1.**
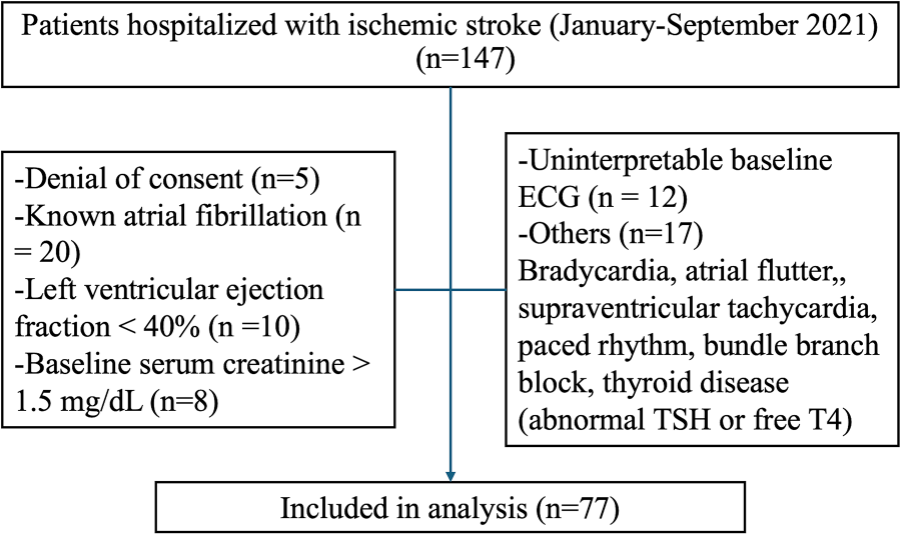
Flow diagram of study cohort selection.

A total of 77 patients with complete clinical, ECG, echocardiographic, at least 24 h Holter recordings, and laboratory data were included in the final analysis (Figure 1. Flowchart). Follow-up data regarding incident AF and recurrent ischemic stroke were obtained retrospectively via the institutional electronic health records over a 3-year period

An a priori power analysis indicated that a sample size of at least 102 patients (51 per group) would be required to detect a clinically meaningful difference in AF incidence with 80% power and α = 0.05. Due to time-limited prospective enrollment and strict eligibility criteria, only 77 patients were ultimately included.

## ECG Acquisition and Scoring

Standard 12-lead ECGs were recorded 3 to 5 days after the index stroke event, following initial stabilization. All ECGs were independently reviewed by two cardiologists blinded to patient outcomes. Measurements were performed manually using digital calipers. Discrepancies were resolved by consensus. Interobserver agreement was evaluated in a random 20% sample and showed high concordance (Cohen's κ = 0.86 for categorical variables; intraclass correlation coefficient [ICC] = 0.91 for continuous variables).

The SIMP3L2E score includes six ECG components: PR interval >200 ms (3 points), QTc prolongation (>450 ms in men or >460 ms in women; 2 points), left axis deviation (QRS axis < –30°; 2 points), presence of ≥1 premature atrial contraction (PAC) on ECG (4 points), left ventricular hypertrophy (LVH) based on standard voltage criteria (4 points), and QRS duration ≥120 ms (3 points). The total score ranges from 0 to 18. A score ≥12 was defined as high risk, based on the original derivation study.^7^

Although the SIMP3L2E score was not available at the time of data collection, all six required ECG parameters were routinely recorded as part of the initial stroke workup. The score was retrospectively calculated in this prospectively followed cohort, serving as an external validation in a real-world stroke population.

Supraventricular ectopy was defined as the presence of ≥500 PAC, supraventricular runs (SVRUN), or brief AF episodes (<30 s) on baseline 24 h Holter monitoring. These arrhythmias were combined into a binary composite variable (present/absent) and included in all predictive analyses as an indicator of atrial electrical instability.

## Outcomes and Statistical Analysis

The primary outcome was the development of incident AF during the three-year follow-up, confirmed through standard or Holter ECG recordings. The secondary outcome was recurrent ischemic stroke, diagnosed clinically and confirmed by neuroimaging.

Statistical analyses were conducted using SPSS version 27.0 (IBM Corp., Armonk, NY, USA). Continuous variables were presented as mean ± standard deviation (SD) or median (interquartile range, IQR), and compared using Student's t-test or Mann–Whitney U test, depending on distribution. Categorical variables were reported as counts and percentages, and compared using Chi-square or Fisher's exact test. Variables with p < .10 in univariate analysis or deemed clinically relevant were included in multivariate logistic regression models. Predictive performance was assessed using receiver operating characteristic (ROC) curves and area under the curve (AUC) values. A two-sided p-value <.05 was considered statistically significant.

## Results

A total of 77 patients were included in the final analysis. According to the SIMP3L2E score, 27 patients (35.1%) were classified as high risk (score ≥12), while 50 patients (64.9%) were in the lower-risk group (score <12). Patients in the high-risk group were significantly older than those in the lower-risk group (76.6 ± 14.4 vs 65.9 ± 14.4 years, p = .001). No significant differences were observed between the groups in terms of sex, BMI, blood pressure, smoking status, comorbidities (hypertension, diabetes mellitus, coronary artery disease, or previous stroke), or cardiovascular medication use (Table 1).

**Table 1.** Baseline Characteristics of the Cohort.

A. Demographics and Vital Signs	Simple Score ≥12 (n = 27)	Simple Score <12 (n = 50)	p-value
Age, years	76.6 ± 14.4	65.9 ± 14.4	.001
BMI, kg/m²	28.2 ± 3.0	27.8 ± 3.0	.632
Systolic BP, mm Hg	128.9 ± 16.9	129.9 ± 16.9	.814
Diastolic BP, mm Hg	77.5 (70-80)	70 (70-90)	.664
Male sex, n (%)	20 (74.1%)	25 (50.0%)	.054
**B. Clinical History**			
Smoking, n (%)	4 (14.8%)	15 (30.0%)	.174
Hypertension, n (%)	13 (48.1%)	27 (54.0%)	.641
Diabetes mellitus, n (%)	9 (33.3%)	17 (34.0%)	1.000
Prior stroke, n (%)	3 (11.1%)	7 (14.0%)	1.000
Coronary artery disease, n (%)	6 (22.2%)	6 (12.0%)	.325
CPD, n (%)	1 (3.7%)	2 (4.0%)	1.000
**C. Medications at Baseline**			
ARB, n (%)	2 (7.4%)	4 (8.0%)	1.000
ACE, n (%)	4 (14.8%)	9 (18.0%)	1.000
Beta blocker, n (%)	2 (7.4%)	2 (4.0%)	.609
CCB, n (%)	3 (11.1%)	6 (12.0%)	1.000
Clopidogrel, n (%)	2 (7.4%)	2 (4.0%)	.609
Acetylsalicylic acid, n (%)	4 (14.8%)	9 (18.0%)	1.000
Oral antidiabetic, n (%)	2 (7.4%)	10 (20.0%)	.197

BMI, body mass index; BP, blood pressure; CPD, chronic pulmonary disease; ARB, angiotensin receptor blocker; ACE, angiotensin-converting enzyme inhibitor; CCB, calcium channel blocker.

Values are presented as mean ± SD, median (interquartile range), or number (%).

Laboratory analyses showed lower median serum creatinine levels in the high-risk group (0.7 vs 0.8 mg/dL, p = .014) and a slightly higher white blood cell count (7.4 vs 7.0 × 10⁹/L, p = .046), though values remained within normal limits. No significant differences were noted in lipid profiles, liver enzymes, hemoglobin, or other hematological markers (Table 2).

**Table 2.** Laboratory, ECG, Echocardiography, and Holter Findings at Baseline.

A. Laboratory Values	Simple Score ≥12 (n = 27)	Simple Score <12 (n = 50)	p-value
Glucose, mg/dL	141.7 ± 73.3	143.9 ± 73.3	.896
Creatinine, mg/dL	0.7 (0.6-0.8)	0.8 (0.6-1.0)	.014
ALT, U/L	14.0 (10.3-19.0)	11.0 (7-13.0)	.987
Albumin, g/dL	3.65 ± 0.30	3.58 ± 0.30	.516
HDL, mg/dL	43.0 ± 8.5	41.5 ± 8.5	.472
Triglycerides, mg/dL	126 (100.3-177)	92 (84-259)	.849
Total cholesterol, mg/dL	184.5 (154.8-200.5)	206.0 (159-206)	.353
LDL, mg/dL	108.9 ± 26.6	107.5 ± 26.6	.824
Sodium, mmol/L	138.3 ± 2.4	138.6 ± 2.4	.637
Potassium, mmol/L	4.34 ± 0.37	4.29 ± 0.37	.632
WBC, × 10³/uL	7.4 (6.2-10.0)	7.0 (5.5-8.1)	.046
Hemoglobin, g/dL	13.5 ± 2.0	13.5 ± 2.0	.964
Platelets, × 10³/uL	222.9 ± 69.5	242.6 ± 69.5	.231
Lymphocytes, × 10³/uL	2.0 (1.6-2.3)	1.7 (1.4-2.1)	.221
Neutrophils, × 10³/uL	4.41 ± 2.07	5.13 ± 2.07	.400
**B.ECHO, ECG and Holter parameters**			
Ejection fraction, %	60(55-65)	60(55-65)	.377
LA diameter, mm	36.0 ± 3.7	35.1 ± 3.7	.395
Heart rate, bpm	80.0 ± 14.8	75.9 ± 14.8	.237
P-wave amplitude, mV	0.095 (0.08-0.11)	0.08 (0.06-0.12)	.082
PR interval, ms	174.6 ± 22.2	158.6 ± 22.2	.037
QRS duration, ms	95.6 ± 11.5	95.1 ± 11.5	.953
QTc duration, ms	458.9 ± 38.8	445.3 ± 38.8	.123
QT duration, ms	401.2 ± 38.2	399.8 ± 38.2	.879
Supraventricular ectopy (Holter), n (%)	19 (70.4%)	12 (24.0%)	<.001
AF episodes (Holter), n (%)	2 (7.4%)	3 (6.0%)	1.000

ALT, alanine aminotransferase; HDL, high-density lipoprotein cholesterol; LDL, low-density lipoprotein cholesterol; WBC, white blood cell; LA, left atrium; HR, heart rate; AF, atrial fibrillation; SVRUN, supraventricular run; QTc, corrected QT interval; ECG, electrocardiography; ECHO, echocardiography.

Values are presented as mean ± SD, median (interquartile range), or number (%).

Among ECG parameters, PR interval was significantly longer in the high-risk group (174.6 ± 22.2 vs 158.6 ± 22.2 ms, p = .037), while QRS duration and QTc interval did not differ significantly. Echocardiographic findings, including LVEF and left atrial diameter, were comparable between the groups. Supraventricular ectopy-defined as the presence of ≥1 PAC, SVRUN, or brief AF episodes on Holter-was significantly more frequent in the high-risk group (70.4% vs 24.0%, p < .001). Overt AF on Holter was infrequent and showed no significant difference (7.4% vs 6.0%, p = 1.000) (Table 2).

During the 3-year follow-up period, new-onset AF was detected in 12 patients (15.6%). AF occurred in 6 patients in each group: 6/27 (22.2%) in the high-risk group versus 6/50 (12.0%) in the low-risk group (p = .325). Recurrent ischemic stroke occurred in 9 patients (11.7%) with no significant difference between the groups (Table 3).

**Table 3.** Clinical Outcomes During 3-Year Follow-Up.

Clinical Outcome	Simple Score ≥12 (n = 27)	Simple Score <12 (n = 50)	p-value
AF developed, n (%)	6 (22.2%)	6 (12.0%)	.325
Stroke recurrence, n (%)	4 (14.8%)	5 (10.0%)	.712

Statistical analysis performed with Chi-square or Fisher's exact test.

## Predictive Analyses

In univariate analysis, only supraventricular ectopy was significantly associated with incident AF (p = 0.002). Age (p = .837), PR interval (p = .216), SIMP3L2E ≥ 12 (p = .325), hypertension (p = .883), and diabetes mellitus (p = .741) were not significant predictors (Table 4). In multivariate logistic regression, supraventricular ectopy remained the only independent predictor of incident AF (OR: 0.092; 95% CI: 0.016-0.538; p = .008), while age, PR interval, and SIMP3L2E score were not retained in the model. The model showed good fit (Nagelkerke R² = 0.257; Hosmer–Lemeshow p = .212).

**Table 4.** Regression Analysis for Predictors of Atrial Fibrillation During 3-Year Follow-Up.

Variable	Univariate OR (95% CI)	p-value	Multivariate OR (95% CI)	p-value
Age, per year	0.997 (0.941-1.055)	.909	1.006 (0.952-1.062)	.837
PR duration, per ms	0.987 (0.962-1.014)	.342	0.987 (0.963-1.012)	.307
Simple Score ≥12	0.994 (0.173-5.713)	.995	1.196 (0.234-6.109)	.830
Supraventricular ectopy (Holter)	0.091 (0.015-0.558)	.010	0.092 (0.016-0.538)	.008
Hypertension	0.711 (0.150-3.356)	.666	–	–
Diabetes mellitus	2.374 (0.400-14.082)	.341	–	–
Male sex	1.238 (0.264-5.804)	.787	–	–

OR, odds ratio; CI, confidence interval; AEV, atrial ectopic beat; SVRUN, supraventricular run; AF, atrial fibrillation.

**Figure 2.**
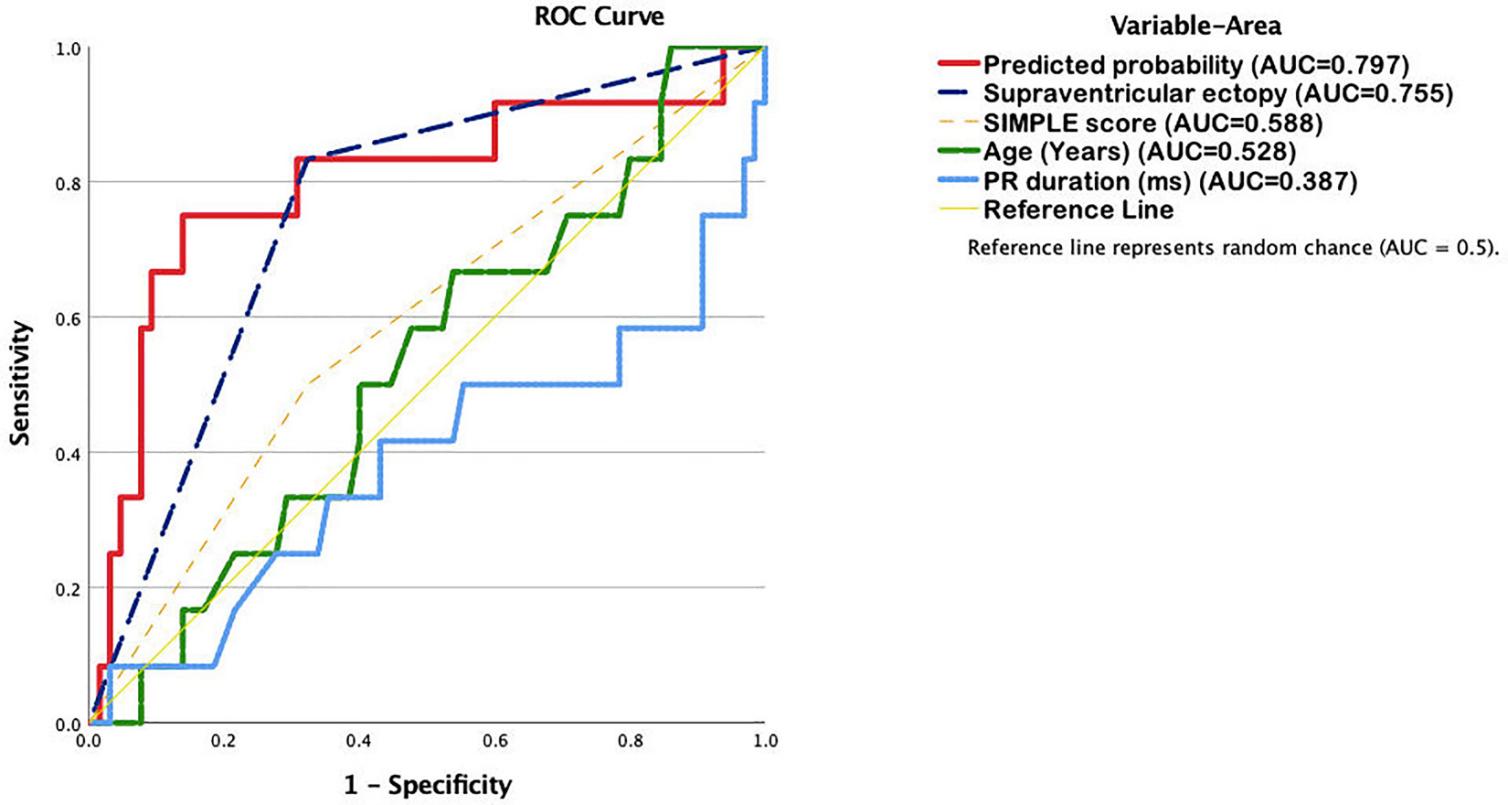
ROC analysis for predictors of AF within 3 years. The multivariate model (AUC = 0.797) showed the best performance, followed by supraventricular ectopy findings on Holter (AUC = 0.755). SIMP3L2E score (AUC = 0.588), age (AUC = 0.528), and PR interval (AUC = 0.387) had lower predictive value. The reference line indicates random prediction (AUC = 0.5).

Receiver operating characteristic (ROC) curve analysis revealed the highest discriminatory performance for the multivariate model (AUC = 0.797), followed by supraventricular ectopy alone (AUC = 0.755). The SIMP3L2E score alone had limited predictive capacity (AUC = 0.588), while age (AUC = 0.528) and PR interval (AUC = 0.387) demonstrated poor discrimination (Figure 2).

## Discussion

In this prospective cohort of ischemic stroke survivors, we externally validated the predictive utility of the SIMP3L2E ECG score for incident AF. While the score alone showed only modest discrimination in this older, comorbid population (AUC = 0.588), its predictive performance improved substantially when combined with Holter-detected supraventricular ectopy. The multivariate model incorporating supraventricular arrhythmic activity yielded the highest discriminatory power (AUC = 0.797), suggesting that short-term ambulatory rhythm monitoring adds significant incremental value beyond static ECG parameters. These findings align with previous studies emphasizing the prognostic relevance of atrial ectopy in cryptogenic stroke and paroxysmal AF detection.^5,7^

The limited standalone performance of the SIMP3L2E score in our cohort contrasts with its original validation in a general Japanese population, where it demonstrated an AUC of 0.73-0.75 for predicting incident AF.^7^ This discrepancy likely reflects fundamental differences in patient characteristics. While the original derivation cohort included middle-aged, community-dwelling adults without recent cardiovascular events, our population was older and composed exclusively of patients recently hospitalized for acute ischemic stroke. In particular, patients in the high-risk group (SIMP3L2E ≥ 12) were, on average, more than 10 years older than those in the lower-score group. Age-related atrial remodeling, subclinical fibrosis, and multimorbidity-hallmarks of atrial cardiomyopathy-may have increased the baseline risk of AF across the cohort, thus attenuating the incremental predictive value of ECG-based markers.^10^ Although age was not a statistically significant predictor in our multivariate model, the observed effect size and confidence interval suggest a possible trend that may become more evident in larger samples.

Our findings underscore the clinical importance of supraventricular ectopy in identifying post-stroke patients at high risk for developing incident AF. This arrhythmic activity-detected by short-term Holter monitoring-was the only independent predictor of new-onset AF and demonstrated superior discriminatory ability compared to traditional ECG markers. These results align with prior research indicating that frequent PACs and supraventricular runs serve as early indicators of atrial electrical instability, which may precede the onset of overt AF.^5^ In the post-stroke setting, where the risk of cardioembolic recurrence is elevated and AF often goes undiagnosed, the detection of such subclinical arrhythmic activity could provide a critical window for timely intervention. By integrating short-term rhythm monitoring into routine post-stroke care, clinicians could better identify high-risk patients and initiate preventive strategies, such as prolonged monitoring or empiric anticoagulation, before AF develops.^4,5^

When compared to established clinical scores such as CHADS₂ and CHA₂DS₂-VASc, which were originally developed for thromboembolic risk stratification in patients with known AF, the SIMP3L2E score offers a distinct advantage by relying solely on ECG parameters. However, the SIMP3L2E score's predictive value for incident AF was limited in our cohort (AUC = 0.588), which is lower than what has been reported for more stroke-specific tools like AS5F and CHASE-LESS. These latter scores incorporate additional clinical variables, such as stroke severity and age-related factors, and show improved performance in predicting AF in post-stroke populations.^8,9^ While ECG-based scores, like SIMP3L2E, provide a simple, non-invasive, and cost-effective tool, they may require recalibration for older or high-risk stroke populations to improve predictive accuracy. The simplicity and objectivity of ECG-based scoring are still advantages, particularly in resource-limited settings, where more complex tools like long-term monitoring devices may not be readily available.

These observations resonate with the findings of Grygorowicz et al, who evaluated the performance of the HAVOC and Brown ESUS-AF clinical scores for predicting AF detected via implantable cardiac monitors (ICM) in patients with embolic stroke of undetermined source (ESUS).^11^ In their multicenter cohort of 384 patients, both scores demonstrated moderate predictive power, with the Brown ESUS-AF score achieving an AUC of 72.9% and the HAVOC score yielding an AUC of 68.5%. However, both tools suffered from low sensitivity-55.7% and 45.3%, respectively-resulting in a substantial proportion of true AF cases being missed. This parallels our findings, where the SIMP3L2E score alone also displayed limited discrimination (AUC = 0.588) and failed to independently predict incident AF in multivariate models. Interestingly, Grygorowicz et al further reported that only the Brown ESUS-AF score significantly improved net reclassification index (NRI) and integrated discrimination index (IDI) over the CHA₂DS₂-VASc score, underscoring the limitations of conventional clinical tools in capturing subclinical AF.

These findings have important implications for post-stroke secondary prevention. While the SIMP3L2E score alone may be insufficient to guide long-term rhythm monitoring strategies in older, high-risk patients, its combination with short-term Holter findings-particularly supraventricular ectopy-could help identify individuals who may benefit from extended monitoring or early empirical anticoagulation. Given that standard stroke workups often include 12-lead ECG and at least 24-h Holter ECG, incorporating both static and dynamic rhythm markers into routine evaluation could enhance detection of subclinical AF. This approach may offer a pragmatic and cost-effective method for risk stratification in real-world clinical settings, where access to long-term rhythm monitoring tools such as implantable loop recorders remains limited.

In addition to ECG-based scores, circulating biomarkers have shown promise in predicting incident AF after stroke. Notably, Grygorowicz et al found that patients who developed incidental AF during follow-up had significantly higher NT-proBNP levels compared to those who remained in sinus rhythm.^11^ Similarly, Shiroto and Hagii reported that both NT-proBNP and BNP levels were elevated in patients with paroxysmal AF (PAF), with BNP showing a slight advantage in terms of IDI.^12^ These biomarkers were identified as independent predictors of covert AF, although their moderate diagnostic performance suggests they are better suited as adjunctive tools rather than standalone predictors. While our study did not assess biomarkers, future research could explore the combined use of ECG-based scores and biomarkers to enhance risk stratification strategies in post-stroke care.

Parallel to these findings, machine learning (ML) models have gained traction in AF prediction, including in stroke patients. A meta-analysis by Goh and Bhaskar reviewed ML models in AF prediction, including studies of 246,559 patients, and found an average AUROC of 0.75, indicating moderate success.^13^ Notably, Deep Neural Networks (DNN) and XGBoost (XGB) demonstrated the best performance with ROCs of 0.77 and 0.76, respectively, showing their potential in enhancing AF risk stratification. In our study, while the SIMP3L2E ECG score demonstrated only modest predictive capacity (AUC = 0.588), the combination of this score with supraventricular ectopy detected on Holter ECG provided significantly improved predictive performance (AUC = 0.797). These findings suggest that integrating biomarkers, ECG-based scores, and advanced ML models could offer a more comprehensive approach to AF risk stratification in post-stroke populations, although further validation and refinement of these models are necessary to establish their clinical utility.

## Limitations and Future Perspectives

Several limitations of this study should be acknowledged. First, the relatively small sample size limited the statistical power, as indicated by the modest number of events observed. This highlights the risk of type II error, and the non-significant results, particularly those related to the SIMP3L2E score, should be interpreted with caution. Second, the study was conducted at a single center with strict inclusion and exclusion criteria. This limits the generalizability of the findings to broader stroke populations. Although the cohort was well-characterized, it may not fully reflect the diversity of patients encountered in everyday clinical practice. Third, although patients were prospectively enrolled, follow-up data were collected retrospectively from electronic health records, which may have introduced bias due to missing information or incomplete documentation. In addition, stroke severity data, such as NIHSS scores, were not included in the multivariate analysis, as the primary aim was to assess ECG-based predictors rather than construct a comprehensive clinical risk model.

Another limitation is the potential underperformance of the SIMP3L2E score in comparison to other methods, such as biomarkers and machine learning models. While these tools, including NT-proBNP, BNP, and ML models like DNN and XGB, show promise in AF prediction, they still require further validation in post-stroke populations. The integration of biomarkers and ML models into clinical workflows remains a challenge, and their application in real-world clinical settings requires further refinement and testing.

Future studies should validate the SIMP3L2E score in larger, multi-center cohorts, and further research is needed to assess the clinical utility of combining ECG-based scores, biomarkers, and ML models for more accurate AF prediction in stroke patients. Prospective trials exploring the impact of integrating short-term rhythm monitoring into routine post-stroke care could provide insight into whether this approach reduces AF-related complications and improves patient outcomes.

## Conclusion

In this prospective cohort of ischemic stroke survivors, we validated the predictive utility of the SIMP3L2E ECG score for new-onset atrial fibrillation (AF) and recurrent stroke. While the SIMP3L2E score alone showed modest predictive power, combining it with supraventricular ectopy detected by Holter ECG improved its discriminative ability significantly, highlighting the importance of integrating dynamic rhythm monitoring in stroke care. These findings suggest that ECG-based scoring can be a valuable, non-invasive tool for identifying high-risk post-stroke patients, especially when combined with short-term rhythm monitoring.

Despite the promising results, the relatively small sample size and single-center design limit the generalizability and statistical power of our study. Future research should validate the SIMP3L2E score in larger, multi-center cohorts and explore its combination with other clinical and imaging parameters to enhance its utility in clinical practice. Additionally, prospective trials are needed to determine whether early detection of subclinical AF via short-term monitoring can reduce AF-related complications and improve long-term stroke outcomes.
